# Assembly strategies for polyethylene-degrading microbial consortia based on the combination of omics tools and the “Plastisphere”

**DOI:** 10.3389/fmicb.2023.1181967

**Published:** 2023-04-17

**Authors:** Chengxiao Zhang, Yulin Mu, Taihua Li, Feng-Jie Jin, Chun-Zhi Jin, Hee-Mock Oh, Hyung-Gwan Lee, Long Jin

**Affiliations:** ^1^College of Biology and the Environment, Co-Innovation Centre for Sustainable Forestry in Southern China, Nanjing Forestry University, Nanjing, China; ^2^Cell Factory Research Centre, Korea Research Institute of Bioscience & Biotechnology, Daejeon, Republic of Korea

**Keywords:** polyethylene biodegradation, microbial consortia, plastisphere, microplastics, nanoplastics, omics

## Abstract

Numerous microorganisms and other invertebrates that are able to degrade polyethylene (PE) have been reported. However, studies on PE biodegradation are still limited due to its extreme stability and the lack of explicit insights into the mechanisms and efficient enzymes involved in its metabolism by microorganisms. In this review, current studies of PE biodegradation, including the fundamental stages, important microorganisms and enzymes, and functional microbial consortia, were examined. Considering the bottlenecks in the construction of PE-degrading consortia, a combination of top-down and bottom-up approaches is proposed to identify the mechanisms and metabolites of PE degradation, related enzymes, and efficient synthetic microbial consortia. In addition, the exploration of the plastisphere based on omics tools is proposed as a future principal research direction for the construction of synthetic microbial consortia for PE degradation. Combining chemical and biological upcycling processes for PE waste could be widely applied in various fields to promote a sustainable environment.

## 1. Introduction

Plastic pollution remediation is always a global environmental protection issue. As the most commonly used material, plastic products are, however, increasingly in demand and used in all fields globally along with socio-economic development due to their excellent characteristics such as durability, low cost and convenience, and it is predicted that the global production of plastic products will be over 26 billion tons by 2050 (Plastics Europe, 2021; Zeenat et al., 2021; Liu et al., 2022b). Approximately 80% of the world’s 400 million tons of plastic waste is dumped in landfills or discarded directly into the environment (Tejaswini et al., 2022). And the most common types of plastic waste are divided into two categories according to their thermal properties: thermoplastics and thermosetting plastics (Canopoli et al., 2018). Thermoplastics are types of linear-chain polymer compounds that have plasticity at a certain temperature, the most common of which are polyethylene (PE), polyvinyl chloride (PVC), polypropylene (PP), and polystyrene (PS). On the other hand, thermosetting plastics, such as polyurethane (PUR), cannot be melted due to irreversible thermal chemical changes (Amobonye et al., 2021). The backbones of thermosetting polymers are highly crosslinked by heteroatoms, making it easy for fracture to occur at ester or amide bonds. In contrast, the junctions of the primary chains in thermoplastics, which mostly comprise carbon atoms, render them more resistant to deterioration (Zheng et al., 2005). The majority of plastic waste is sent to landfill or discharged directly into the environment where it undergoes a very sluggish natural degradation process, which for PE specifically, the most inert polyolefin plastic, the half-lives were estimated to vary from decades to centuries (Chamas et al., 2020).

**FIGURE 1:**
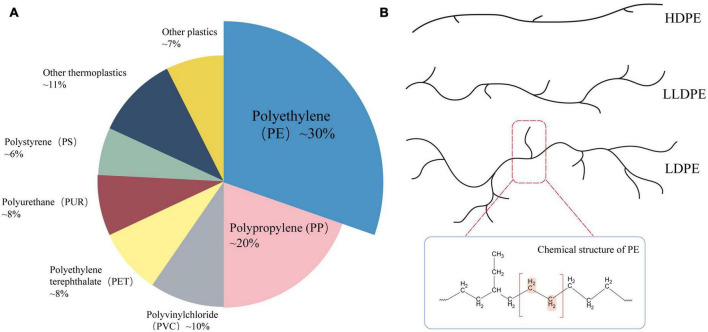
**(A)** Market share distribution of plastic types in 2021 (Plastics Europe, 2021); **(B)** common types of polyethylene (PE) and their molecular structure.

As the primary C–C-chain polymer (Figure 1), PE is currently the most extensively used plastic type, accounting for approximately 38% of the market share (Danso et al., 2019). PE is a thermoplastic made of ethylene that has been through high-pressure polymerization (Kumar Sen and Raut, 2015). According to different densities, branching degrees, and the availability of surface functional groups, PE can be divided into low-density polyethylene (LDPE), high-density polyethylene (HDPE), linear low-density polyethylene (LLDPE), etc. (Restrepo-Flórez et al., 2014). PE is widely utilized in cling film, commercial plastic bags, pharmaceutical and food packaging films, and other consumer and manufacturing industries, because of its desirable properties, such as non-toxicity, tastelessness, high tensile strength, low permeability, and durability (Shah et al., 2008; Liu et al., 2021). Due to its large molecular weight, stable chemical structure, high hydrophobicity, crystallinity, and limited number of functional groups required for biodegradation, PE is the plastic polymer most resistant to degradation, this means that polyethylene debris has lingered in the marine and other ecosystems for two decades and is extremely resistant to degradation (Kyaw et al., 2012; Dey et al., 2020; Krause et al., 2020). In addition, PE is the most prevalent municipal solid waste (MSW), and accounts for a high proportion of plastic waste in the environment (Zhou et al., 2014). Therefore, understanding the mechanisms of PE degradation and devising effective and environmentally friendly methods for PE degradation could provide ideas and data to help to mitigate our ever-worsening plastic pollution on a massive scale.

Current research indicates that the decomposition of plastic waste, such as PE, in landfills and other ecosystems is predominantly accomplished via physicochemical (abiotic) degradation and biodegradation (Dimassi et al., 2022). Physical degradation, such as cracking, embrittlement, and spalling, typically modifies the primary structures of polymers; whereas chemical degradation primarily modifies the molecular structures of polymers, such as breaking bonds or the oxidation of long polymer chains to produce compounds with low molecular weights (Andrady, 2011; Gewert et al., 2015; Ali et al., 2021). Mechanical degradation (including tidal forces, waves, and erosion), photo-oxidation (such as ultraviolet rays), thermal oxidation (such as incineration, pyrolysis, and gasification), and chemical hydrolysis processes (such as those involving acids, alkalis, and other organic solvents) are the primary physicochemical degradation mechanisms for PE and other plastic waste in the environment (Kyrikou and Briassoulis, 2007). Among them, photodegradation and thermo-oxidative degradation are the most common mechanisms of PE degradation in the environment (Canopoli et al., 2020). Through a variety of physical and chemical processes, large-sized plastic waste is broken down into plastic debris and micro/nanoplastics, which are then more easily digested and consumed by microorganisms (Taghavi et al., 2021b; Singh Jadaun et al., 2022). Researchers have become increasingly interested in the biodegradation of plastic in recent years due to the high efficiency, availability, and eco-friendliness of this approach (Khatoon et al., 2017). Deepening our understanding of the natural microbial communities that arise in the plastisphere may lead to novel approaches for the development of PE-degrading consortia. The key to the construction of efficient, stable, and controllable consortia is the design, which needs to ensure that the microorganisms in these communities interact synergistically. Certain technologies, such as next-generation sequencing technologies like metagenomic sequencing, can be used to determine the microbial community structure of the plastisphere (Gilmore et al., 2019; San León and Nogales, 2022).

This study focused on PE, the most prevalent plastic polymer. We briefly examined its characteristics and biodegradation mechanisms, as well as the current research gaps and recent advances in PE biodegradation. Then, we explored the advantages of using microbial functional consortia and their potential for PE pollution remediation compared to single strains. Lastly, we emphatically investigated the prospect of combining the plastisphere concept and metagenomics to inform the construction of functional microbial consortia.

## 2. Biodegradation of PE

Polyethylene (PE) biodegradation occurs via bacteria, fungi, algae, and other microorganisms that adhere to the plastic’s surface and consume it for growth and reproduction (Taghavi et al., 2021a). Microorganisms face a considerable challenge with PE due to its high hydrophobicity, large molecular size, and lack of reactive functional groups in its polymer backbone (Cowan et al., 2022). Numerous microorganisms (including bacteria, fungi, actinomycetes, and even some algae) that are capable of degrading PE polymers in various ecosystems (including oceans, soil, farmland, animal manure, compost, landfills, sewage, etc.) have been discovered using culture-dependent or culture-independent techniques (Dey et al., 2020). Several microbial enzymes associated with plastic biodegradation, particularly those involved in PE biodegradation, such as laccase, manganese peroxidase, lignin peroxidase, and alkane hydroxylase (AH), have also been found to play crucial roles (Wei and Zimmermann, 2017). It is worth noting that laccase and peroxidase (manganese peroxidase and lignin peroxidase) are also key enzymes in lignin degradation (Kavitha and Bhuvaneswari, 2021). Lignin as a complex amorphous aromatic biopolymer, which is connected by carbon–carbon bonds and ether bonds. Additionally, lignin and PE have partial similarities in terms of their structural and physicochemical properties, as they both have the characteristics of high hydrophobicity and high molecular weight. Thus, the enzymes required for the biodegradation of lignin, as a recalcitrant natural polymer, are similar to those required for the degradation of PE in that their degradation relies on the involvement of oxidoreductases to catalyze oxidation and generate free radicals to drive depolymerization (Kumar and Chandra, 2020; Daly et al., 2021).

### 2.1 Mechanisms of plastic degradation by microorganisms

**FIGURE 2:**
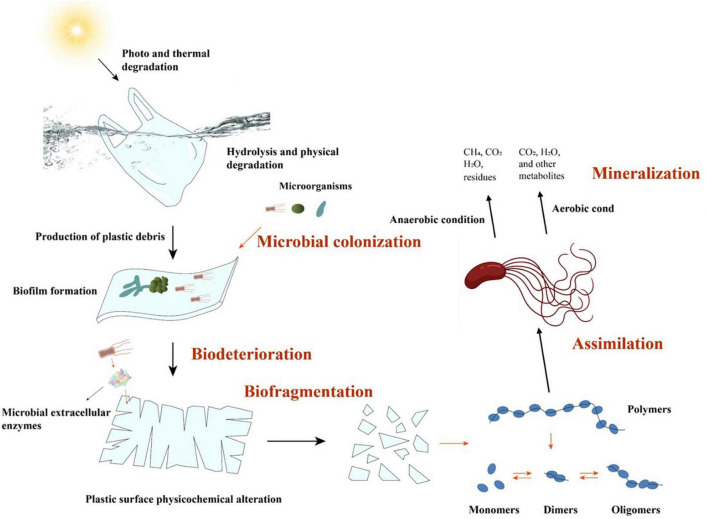
A diagram of the microbial degradation processes of polyethylene (PE).

The microbial degradation of plastic polymers, including PE, generally involves five basic stages (Figure 2): microbial colonization, biodeterioration, biofragmentation, assimilation, and mineralization (Gu, 2003).

(i) The initial phase involves the colonization of microorganisms on the surface of the plastic material. During this period, extracellular polysaccharides or biosurfactants allow microbes to cluster more easily and cling to the hydrophobic surface of the plastic material (Kavitha and Bhuvaneswari, 2021; Ji et al., 2022). The formation of biofilms on polymer surfaces not only is conducive to the colonization of microorganisms, but also severely damages the surfaces, enabling the addition of functional groups.

(ii) Typically, the microorganisms that colonize the surface of a plastic material are the cause of biodeterioration. Through the production of enzymes (such as lipase or dehydrogenase) or other secretions, these colonizing microbes penetrate polymer materials and subsequently generate interstices and cracks (Thakur et al., 2018; Rana et al., 2022). Additionally, some filamentous microorganisms (such as actinomycetes and molds) can deteriorate plastic matrices via their filamentous network structures (Sánchez, 2020). Moreover, the production of acids and alkalis during microbial metabolic processes may, in some cases, intensify the erosion of polymer surfaces (Ali et al., 2021).

(iii) Biofragmentation is the lytic process by which microorganisms convert macromolecular polymers into oligomers, dimers, or monomers via the action of enzymes or free radicals (Ghatge et al., 2020). Since polymers with macromolecular structures cannot be directly absorbed or utilized by microorganisms, they have to be disintegrated into smaller products that are suited to cell absorption and subsequent metabolism outside the cell (Lucas et al., 2008). PE, the main C–C-chain polymer with a high molecular weight, can only be supplied to microbial cells for further metabolism after being broken into oligomers of 10–50 carbon atoms (Krueger et al., 2015).

(iv) When polymers have been degraded into oligomers with low molecular weights (< 600 Da), microbes can easily absorb these oligomers via assimilation (Du et al., 2021). Finally, the polymers are mineralized under aerobic or anaerobic conditions through a range of metabolic pathways and to produce metabolites such as CO_2_, CH_4_, and H_2_O (Pathak and Navneet, 2017).

### 2.2 Bacteria involved in PE degradation

Several studies have found that bacteria can inhabit plastic-polluted environments (soil, water, etc.) by forming biofilms on the surfaces of discarded plastic debris, meaning that these microbial communities may be able to colonize plastic surfaces more effectively (Basili et al., 2020; Vaksmaa et al., 2021). The colonizing bacteria secrete the corresponding plastic-degrading enzymes, which catalyze the decomposition of the polymers into oligomers, dimers, and monomers through a combination of enzymes and finally mineralize them into other products, such as CO_2_ and H_2_O. Recent research has shown that *Bacillus* (Samanta et al., 2020), *Enterobacter* (Ren et al., 2019; Sarker et al., 2020), *Brevibacterium* (Hadad et al., 2005; Dwicania et al., 2019b), *Pseudomonas* (Hou et al., 2022), *Arthrobacter* (Balasubramanian et al., 2010), *Acinetobacter* (Montazer et al., 2018; Zhang et al., 2023), and other prevalent bacterial genera have the ability to degrade PE (Table 1). Notably, *Bacillus* spp. and *Pseudomonas* spp. also play key roles in the degradation of other plastic polymers, including polyethylene terephthalate (PET), PS, PP, and PUR (Auta et al., 2018; Roberts et al., 2020; Ganesh Kumar et al., 2021; Roy et al., 2021). In addition, bacteria that survive in extreme conditions, such as those that are thermophilic, acidophilic/alkaline, halophilic, radiation-resistant, etc., have significant potential for degrading PE (Atanasova et al., 2021). Mouafo Tamnou et al. (2021) utilized *Pseudomonas aeruginosa* to degrade PE under the conditions of an acidic environment (pH = 5) and a temperature of 44°C, and the weight loss rate of PE reached 6.25% after 30 days of culture. Hadad et al. (2005) recovered a thermophilic bacterial strain (*Brevibacillus borstelensis* 707) from soil and demonstrated that it reduced the weight of LDPE by 11%. Although previous studies have revealed many bacteria with PE-degradation functions, these bacteria have been determined to largely belong to the genera *Bacillus* and *Pseudomonas*; however, with the development of isolation and cultivation technologies, additional PE-degrading bacterial resources may still be discovered.

**TABLE 1:** Bacterial strains have been reported with polyethylene degradation capacity.

Species	Sources	Culture time	Plastic type	Weight loss	Other methods	References
*Stenotrophomonas* sp., *Achromobacter* sp.	Waste dumpsite, drilling fluid	100 days	LDPE beads	7.45%, 7.54%	SEM, AFM, FTIR	Dey et al., 2020
*Lysinibacillus* sp.	Soil	28 days	Polyethylene films	7.50%	SEM, GC-MS, FTIR, XRD, crystallinity change	Jeon et al., 2021
*Bacillus subtilis*	Microbial culture collection	30 days	LDPE films	9.26%	FTIR	Vimala and Mathew, 2015
*Pseudomonas knackmussii*, *Pseudomonas aeruginosa*	Activated sludge or sewage water sample	56 days	LLDPE films	5.95 ± 0.03%, 3.62 ± 0.32%	FTIR, SEM, WCA, AFM	Hou et al., 2022
*Bacillus cereus*, *Bacillus gottheilii*	Sediment	40 days	PE powder	1.6%, 6.2%	SEM, FTIR	Auta et al., 2017
*Micrococcus luteus*	Cow dung	90 days	HDPE films	3.85%	SEM, EDX, FTIR	Gupta et al., 2022
*Microbulbifer hydrolyticus*	Marine pulp mill wastes	30 days	LLDPE particles	–	SEM, FTIR	Li et al., 2020
*Alcaligenes faecalis*	Sea water	70 days	Polyethylene bags	47.36%	FTIR, SEM, XRD, AFM	Nag et al., 2021
*Bacillus* sp.	Plastic waste polluted site	30 days	LDPE bags	6.68 ± 0.59%	FTIR, SEM, GC-MS	Kavitha and Bhuvaneswari, 2021
*Pseudomonas* sp.	Municipal solid waste dumping ground soil	45 days	LDPE films	5 ± 1%	SEM, AFM, tensile strength	Tribedi and Sil, 2013
*Marinobacter* sp. H-244, *Marinobacter* sp. H-246, *B. subtilis*	Seawater and sediment	90 days	LDPE films	1.46%, 1.68%, and 1.54%	SEM, AFM, FTIR, carbon content analysis, TGA, GC-MS	Khandare et al., 2022
*Alcanivorax* sp.	Marine plastic debris	34 days	Pristine and weathered LDPE pellets and films	2.4% (weathered LDPE films)	FTIR, GPC, stable isotope analysis, oxygen species (ROS) analysis	Zadjelovic et al., 2022
*Alcanivorax borkumensis*	The “plastisphere” from marine ecosystem	80 days	LDPE films	3.4%	FTIR	Delacuvellerie et al., 2019
*Kocuria palustris*, *Bacillus pumilus*, *B. subtilis*	Pelagic waters	30 days	Pieces of polyethylene bags	1 ± 0.033%, 1.5 ± 0.038%, 1.75 ± 0.06%	SEM, FTIR	Harshvardhan and Jha, 2013
*Paenibacillus* sp.	Landfill	3 months	polyethylene bags	30.8% (chemical treatment)	FTIR, SEM	Bardají et al., 2019
*Exiguobacterium* sp.	Plastic dumped soil	90 days	LDPE films (Additive free)	5.70? ± ?0.7%	SEM, FTIR, XRD, total carbon analysis	Maroof et al., 2022
*Ralstonia* sp., *Bacillus* sp.	Municipal waste landfill	180 days	LDPE sheets	39.2%, 18.9%	Microscale analysis, FTIR	Biki et al., 2021
*Rhodococcus ruber*	Polyethylene waste buried soil	30 days	LDPE films	8%	SEM, FTIR	Gilan et al., 2004
*Nocardia* sp., *Streptomyces* sp., *Rhodococcus* sp.	Landfills and soil rich in plastic waste	60 days	LDPE films	9.5 ± 0.3%, 5.98 ± 0.72%, 6.23 ± 0.5%	Tensile strength analysis, SEM, FTIR	Soleimani et al., 2021
*Nocardiopsis* sp. mrinalini 9	Healthy leaf	60 days	Polythene bags	22%	BATH assay	Singh and Sedhuraman, 2015

SEM, scanning electron microscope; AFM, atomic force microscope; FTIR, Fourier transform infrared spectroscopy; GC-MS, gas chromatograph- mass spectrometer; WCA: water contact angle; EDX: energy dispersive spectroscopy; GPC: gel permeation chromatography; XRD: X-ray diffraction; TGA: thermogravimetric analysis; BATH, bacterial adhesion to hydrocarbon.

*Actinomycetes*, as a special subgroup of prokaryotes that can form branching hyphae and conidia, are abundant in soil and other habitats (Barka et al., 2016). As highly efficient polymer-degrading agents, *Actinomycetes* have been extensively studied in the degradation of natural polymers, such as lignocellulose (Tan et al., 2022) and natural rubber (Gibu et al., 2020). Regarding the degradation of polymeric polymers by *Actinomycetes*, current research primarily focuses on PET degradation. In the breakdown of PET by *Actinomycetes*, many enzymes that can hydrolyze polyester have been discovered (Wei et al., 2014; Charnock, 2021). *Actinomycetes* also secrete related enzymes during the degradation of PE. In one study, Santo et al. (2013) discovered that the extracellular laccase produced by the actinomycete *Rhodococcus ruber* C208 during PE degradation had greater PE-degradation activity when copper was present. Similar to other bacteria, the actinomycete colonization process is highly impacted by biofilm development. *Actinomycetes* are able to produce extracellular polymeric compounds, such as mucopolysaccharides rich in *N*-acetylglucosamine, which may improve their adhesion to plastic surfaces for later microbial action (Singh and Sedhuraman, 2015). The actinomycetes that have been identified in recent studies as having the ability to degrade PE are predominantly members of the genera *Rhodococcus* (Santo et al., 2013), *Streptomyces* (Soleimani et al., 2021), and *Nocardia* (Table 1; Koutny et al., 2006).

### 2.3 Fungi involved in PE degradation

Fungi play indispensable roles in decomposing recalcitrant substrates and can make recalcitrant polymers, such as plastic, parts of their food web structures, driving the carbon cycle and nutrient regeneration in terrestrial ecosystems (Zeghal et al., 2021). The primary benefit of using fungi over single bacterial strains that can degrade plastic is that fungi grow more broadly in soil and can penetrate polymer surfaces using mycelia, making it easier for them to interact with plastic polymers and subsequently degrade plastic films (Sánchez, 2020). Additionally, fungi can secrete hydrophobic proteins that are capable of adhering to extremely hydrophobic polymer surfaces. These proteins enhance the development of aerial mycelia in filamentous fungi and the adherence of mycelia to hydrophobic polymer surfaces. Due to the high surface activity and strong adhesion properties of these proteins, fungal mycelia can grow more readily than bacteria on hydrophobic plastic surfaces (Piscitelli et al., 2017; Sánchez, 2020). Moreover, the enzymes required for PE degradation, such as laccase, lignin peroxidase, etc., are also found in the fungi-mediated PE breakdown process (Temporiti et al., 2022). Fungi have been shown to be capable of degrading PE, especially strains that belong to the genera *Aspergillus*, *Fusarium*, and *Penicillium* (Taghavi et al., 2021a), among others (Table 2).

**TABLE 2:** Fungal isolates have been reported with polyethylene degradation capacity.

Species	Sources	Culture time	Plastic type	Weight loss	Other methods	References
*Penicillium chrysogenum*, *Fusarium oxysporum*, *Trichoderma brevicompactum*, *Purpureocillium lilacinum*, *Fusarium falciforme*, etc.	Plastic wastes from an abandoned dumpsite	30 days	PE films	–	Respirometry assay, SEM, FTIR	Spina et al., 2021
*Aspergillus clavatus*	Landfill soil	90 days	LDPE films	22%	CO_2_ evolution, FTIR, AFM, SEM	Gajendiran et al., 2016
*Aspergillus tubingensis*, *Aspergillus flavus*	Soil in coastal plastic waste dumping area	30 days	Commercially available HDPE	6.02 ± 0.2%, 8.51 ± 0.1%	FTIR, SEM	Sangeetha Devi et al., 2015
*Penicillium citrinum*	Landfill soil	90 days	LDPE films	38.82%, 47.22% (pretreated with nitric acid)	FTIR, SEM, TGA	Khan et al., 2022
*Alternaria alternata*	Plastic wastes	120 days	PE films	–	SEM, FTIR, XRD, GPC, GC-MS	Gao et al., 2022
*Aspergillus terreus*, *Aspergillus sydowii*	Rhizosphere soil	60 days	PE strips	50.00 ± 4%	Tensile strength analysis, SEM, FTIR	Sangale et al., 2019b
*Trichoderma harzianum*	Dumpsite soil	90 days	Polyethylene bags	23%	SEM, FTIR, NMR	Sowmya et al., 2014
*Trichoderma hamatum*	Plastic from soil along highways	60 days	LDPE and LLDPE films	1.3 ± 0.4% (UV/T60-pretreated LDPE), 3.9 ± 0.5% (γ/T90-pretreated LLDPE)	SEM, FTIR, GPC, TGA	Malachová et al., 2020

NMR, nuclear magnetic resonance spectroscopy.

### 2.4 Enzymes involved in PE degradation

The involvement of enzymes is essential for the microbial degradation of plastic. The molecular weights of plastic polymers are too high for direct uptake by microorganisms; therefore, extracellular enzymes are produced in advance to depolymerize polymers with high molecular weights into oligomers, dimers, monomers, etc., which can be ingested by microbial cells (John, 2019).

As the molecular structures of different plastic polymers vary, identification and further investigation of the specific enzymes for the degradation of different polymers is ongoing. For instance, in the biodegradation of polyethylene terephthalate (PET), it has been found that PETase (an aromatic polyester enzyme) from *Ideonella sakaiensis* 201-F6 can metabolize PET into bis-hydroxyethyl terephthalate (BHET), monohydroxyethyl terephthalate (MHET), terephthalic acid (TPA), and other intermediates, while MHETase (an auxiliary enzyme) further acts on MHET intermediates to convert them into terephthalic acid and ethylene glycol. Both enzymes are found in *I. sakaiensis* secretions and may work cooperatively to degrade PET (Austin et al., 2018). Magnin et al. (2019) demonstrated that a mixture of two enzymes [an efficient amidase (E4143) that hydrolyzes the PUR bonds of molecules with low molar masses and an esterase (E3576) that hydrolyzes aqueous polyester PUR dispersions] could be used to treat thermoplastic polyurethanes, resulting in a synergistic increase in the hydrolysis of PUR bonds.

Polyethylene (PE) has an extremely stable C–C backbone that is more resistant to hydrolysis than those of PET and PUR, which have chemical bonds in their backbones that can be attacked by hydrolases (Yeom et al., 2022). For this reason, certain enzymes and their related mechanisms are still inadequate for PE degradation. As mentioned in the previous section “2.3 Fungi involved in PE degradation”, the main fungal enzymes that have been discovered to be involved in PE degradation are laccase, manganese peroxidase (MnP), and lignin peroxidase (LiP). Gao et al. (2022) analyzed the genomics and transcriptomics of the isolated marine fungus *Alternaria alternata* FB1 with the potential for PE degradation and revealed significant upregulation of the genes that encode 153 enzymes potentially associated with PE degradation, including laccase, peroxidase, and hydroxylase, which further confirmed the ability of some of these enzymes to degrade PE. Laccase is also a potential functional enzyme for PE-degrading bacteria such as *Bacillus*, *Psychrobacter*, etc. (Liu et al., 2022c; Zhang A. et al., 2022). Jeon and Kim (2015) discovered an AH system composed of alkane monooxygenase, rubredoxin, and rubredoxin reductase in *P. aeruginosa* E7. The alkane monooxygenase had LMWPE (low-molecular-weight polyethylene) degradation activity, while the rubredoxin and rubredoxin reductase were indirectly involved in PE degradation through the transfer of relevant electrons. They also identified two alkane monooxygenases (AlkB1 and AlkB2) in *P. aeruginosa* and found that AlkB2 displayed higher transcriptional activity in the presence of LMWPE, thus indicating that AlkB2 was more efficient at degrading LMWPE than AlkB1 (Jeon and Kim, 2016).

**FIGURE 3:**
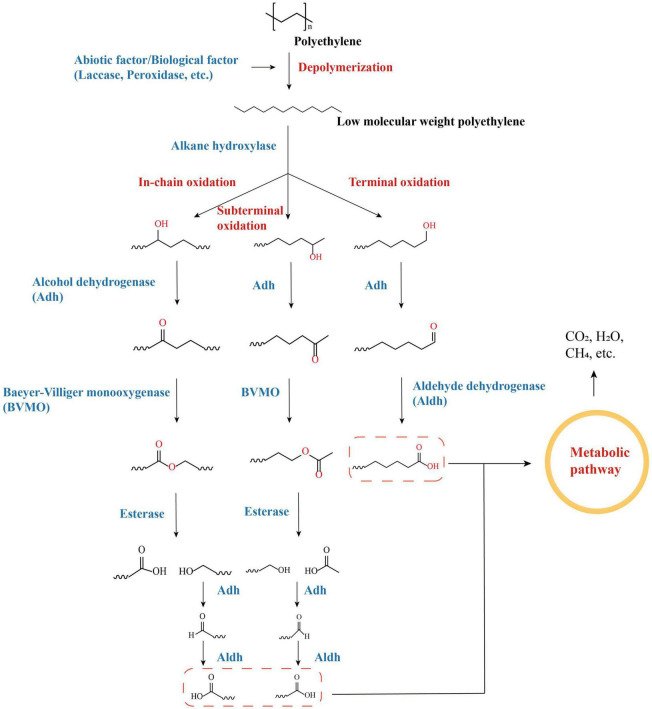
Degradation pathways of PE-derived long-chain linear alkanes by oxidation with alkane hydroxylases (AHs).

Alkane hydroxylases (AHs) are key enzymes in the degradation of alkane compounds such as petroleum, and the genes encoding them have recently been identified in the genomes of a variety of PE-degrading species (Nie et al., 2014; Khalil et al., 2018; Hou et al., 2022). AHs harbor considerable diversity and can differ in terms of substrate ranges and degradation pathways (Ji et al., 2013). Polyethylene polymers with macromolecular weights can be depolymerized to form polymers with low molecular weights, as well as other alkanes, alcohols, and fatty acids with different chain lengths, via a range of abiotic processes (UV irradiation, heat treatment, oxidation, etc.) or enzymatic processes (laccase, peroxidase, etc.) (Zhang Y. et al., 2022). The ends of low-molecular-weight polyethylene can be oxidized by AHs through oxidation processes, including terminal oxidation, subterminal oxidation, etc. As a group of AHs, cytochrome P450 (CYPs) are also candidate enzymes for PE degradation (in addition to the AlkB family that was mentioned previously) and are also key enzymes for PE degradation by PE-degrading bacteria, such as *Rhodococcus* spp. and *Bacillus* spp. (Zampolli et al., 2021; Liu et al., 2022c). The alkanes of different chain lengths that are depolymerized by PE are oxidized by AHs to form alcohols, which are subsequently reacted with a series of cascade enzymes [such as alcohol dehydrogenase (Adh), aldehyde dehydrogenase (Aldh), Baeyer–Villiger monooxygenase (BVMO), and esterase, depending on the hydroxylation terminus] to form alkanoic acids that eventually enter the metabolic pathways of organisms and are consumed (Figure 3; Yeom et al., 2022).

## 3. Biodegradation by microbial consortia

Microbial consortia, as efficient pollutant-degrading agents, have been applied to various environmental remediation fields, such as the degradation of agricultural and forestry residues [cellulose (Wang et al., 2010; Zhao et al., 2017; Du et al., 2018), hemicellulose (Wang et al., 2013; Tabanag et al., 2018; Zhang et al., 2018; Zheng et al., 2020), lignin (Kim et al., 2019; Lu et al., 2019; Zheng et al., 2020; Mendes et al., 2021), etc.], sewage treatment (Halat-Las et al., 2018; Feng et al., 2021), the degradation of persistent organic pollutants (POPs) (Arulazhagan et al., 2010; Li et al., 2021; Xu et al., 2021), and oil pollution treatment (Wu et al., 2017). In recent years, the potential for using microbial consortia in the biodegradation of plastic pollution has been investigated by an increasing number of relevant researchers.

### 3.1. Advantages in the construction of functional PE-degrading microbial consortia

Although many strains capable of degrading PE have been isolated and identified, it takes an incredibly long time for single strains/enzymes to degrade PE and the rate of degradation is exceedingly inefficient due to some of the durability characteristics of PE (Kotova et al., 2021). In addition, studies on the PE degradation products (PE-DPs) that are generated in these biodegradation processes are still in the preliminary stages. Some studies have used gas chromatograph-mass spectrometer (GC-MS) to conduct qualitative analyses and toxicity tests on PE-DPs (Shahnawaz et al., 2016). Studies have confirmed that PE-DPs are mostly composed of fatty acids, plasticizers, benzene, and alcohol; however, it is unclear whether PE-DPs subsequently interfere with microbial growth and metabolic activities (Sangale et al., 2019a). Consequently, these unknown and complicated byproducts may prevent single strains from degrading PE in the latter stages of fermentation. The stable and complicated coexistence of microbial communities arises via evolutionary changes in ecological and biological systems that have occurred over long periods of metabolic activity in the natural environment (Escalante et al., 2015). However, single strains gradually lose their naturally occurring microbial interdependence after isolation and purification, resulting in some attributes being reduced or lost (Ding et al., 2019).

Compared to single strains, microbial consortia are more efficient, robust, and controllable (Qian et al., 2020). (i) Firstly, the biodegradation of plastic polymers is a complex process driven by collaboration between various enzymes and metabolic pathways (Jaiswal et al., 2020). Microbial consortia have more abundant enzyme systems and metabolic activities than single strains (Zhang N. et al., 2022). (ii) Moreover, microbial consortia also create new microenvironments for strains, which may activate some metabolic pathways that are dormant under single-strain culture conditions (Qian et al., 2020). (iii) And then, microbial consortia generally adopt intercellular communication modes, such as quorum sensing (QS), to minimize competition between strains or synergistically regulate gene expression (Duncker et al., 2021). (iv) Concurrently, microbial consortia can execute the division of labor by partitioning metabolic pathways, thereby minimizing the accumulation of byproducts and the metabolic burden of individual strains (Jagmann and Philipp, 2014). (v) From the aspect of stability, microbial consortia that are composed of multifunctional microbes are more robust to environmental perturbations during biodegradation than single-strain cultures (Xu and Yu, 2021). (vi) It is important to note that despite the fact that some strains that are quite prevalent in microbial communities lack degrading capabilities, they may be of indispensable importance in the formation of biofilms on the surface of plastic debris (Giri et al., 2020; Gao and Sun, 2021). (vii) From a long-term perspective, microbial consortia can also effectively use carbon sources and access an expanded spectrum of substrates (Xu and Yu, 2021). Although the majority of plastic debris in the environment consists of mixed plastic, single strains can only target specific substrate polymers. In contrast, multifunctional consortia that are composed of diverse microorganisms can cope with plastic waste containing various types of polymers more effectively due to their vast biodegradation potential (Skariyachan et al., 2022).

### 3.2. Current approaches and applications for construction of synthetic microbial consortia in PE degradation

Current research on the development of synthetic microbial consortia has generally followed two traditional approaches: “top-down” and “bottom-up” (Liang et al., 2022). Top-down techniques involve modification of environmental factors and continual enrichment and serial dilution of natural microbial communities derived from the environment, from which key microbial populations can be identified and stable PE -degrading consortia can ultimately be obtained (Lin, 2022). On the other hand, bottom-up techniques combine isolated strains and/or engineered microorganisms from the same or different sources, depending on their characteristics, and optimize them via appropriate adjustments to construct the desired functional synthetic consortia (Ding et al., 2019).

Recently, some researchers have attempted to construct microbial consortia/co-cultures by mixing single strains to achieve the more efficient degradation of PE, and part investigations on applications of microbial consortia in PE degradation are summarized in Table 3. Park and Kim (2019) combined *Bacillus* sp. and *Paenibacillus* sp. strains that were isolated from landfill sediments and had PE -degradation capabilities to construct a mixed bacterial consortium that, after 60 days of incubation, degraded up to 14.7% of PE microparticles. Skariyachan et al. (2016, 2021) isolated PE degrading strains from a plastic waste disposal area and cow manure, respectively, and then constructed PE degrading microbial consortia that achieved higher PE degradation compared to single strains as well as discovering that PE films lost more weight after treatment with the consortia than PE pellets, which is probably owing to the large surface size and thinness of plastic films giving better conditions for the colonization of microbes. Notably, Esmaeili et al. (2013) demonstrated the ability of fungal–bacterial cross-kingdom consortia to co-degrade LDPE films by burying PE films in soil inoculated with *Aspergillus* and *Lysinibacillus* strains. The advantage of using fungal–bacterial cross-kingdom consortia is that fungal hyphae may represent media for the bacterial colonization of soil that can facilitate the migration of bacterial strains beyond what can be accomplished individually. These studies have formed the basis for investigations into positive interactions between bacteria and fungi that have high potential for the bioremediation of plastic pollutants (Massot et al., 2022). Interestingly, Lou et al. (2022) and Ruiz Barrionuevo et al. (2022) studied the gut microbial communities of PE-fed wax worms and their larvae suggesting that the gut microbes of invertebrates and the constructed microbial consortia are capable of consuming and degrading plastic polymers, thus providing a new resource for the development of functional microbial consortia that have the capacity to degrade PE and other types of plastics. In addition, hydrocarbon-degrading bacteria (HCB) are prominent members of natural microbial communities derived from the plastisphere, given the long-term exposure to plastics, suggesting that PE enrichment cultures of microbial communities derived from the plastisphere will provide new potential for PE-degrading microbial communities (Harrison et al., 2014; Delacuvellerie et al., 2022a; Wang et al., 2022). For example, Joshi et al. (2022) screened potential LDPE-degrading marine bacterial communities associated with the plastisphere from plastic debris collected in coastal environments and then isolated, identified, and evaluated potential marine bacterial strains. Finally, they formulated an associated bacterial consortium that reduced the weight of LDPE by 47.07 ± 6.67% within 120 days.

**TABLE 3:** Microbial consortia/co-cultures constructed with capacity for polyethylene degradation.

Composition of strains in microbial consortia	Sources	Culture time	Plastic type	Weight loss	Other methods	References
*Arthrobacter* sp. and *Streptomyces* sp.	Soil plastic film residues	90 days	PE films	–	WCA, FTIR, SEM, CO_2_ evolution	Han et al., 2020
*Bacillus* sp. and *Paenibacillus* sp.	Landfills	60 days	PE microplastic granules	14.70%	SEM, FTIR, GC-MS, thermogravimetric analysis	Park and Kim, 2019
*Pseudomonas aeruginosa* and *Brevibacterium* sp.	Laboratory isolated	30 days	LLDPE stripes	7.31%	FTIR	Dwicania et al., 2019a
*Enterobacter* sp., *Enterobacter cloacae*, and *P. aeruginosa*	Cow dung	160 days	LDPE films	64.25 ± 2%	SEM, EDS, XRD, FTIR, AFM	Skariyachan et al., 2021
*Acinetobacter* sp. strain NyZ450, and *Bacillus* sp. strain NyZ451	*Tenebrio molitor* larvae gut	30 days	PE mulching films	18.74%	SEM, FTIR, HT-GPC	Yin et al., 2020
*Aneurinibacillus aneurinilyticus* btDSCE01, *Brevibacillus agri* btDSCE02, *Brevibacillus* sp. btDSCE03 and *Brevibacillus brevis* btDSCE04	Activated sludge and plastic contaminated soil	140 days	LDPE stripes	58.21? ± ?2%	FTIR, SEM, AFM, EDS, NMR, GC-MS	Skariyachan et al., 2018
*Bacillus licheniformis*, *Paenibacillus woosongensis*, *Vibrio parahaemolyticus*, and *Vibrio fluvialis*	Plastic debris with “Plastispheres”	120 days	LDPE sheets	47.07 ± 6.67%	SEM, AFM, FTIR, NMR, TG-DSC	Joshi et al., 2022
*Bacillus subtilis*, *Lysinibacillus sphaericus*, *Lysinibacillus fusiformis*, *Alcaligenes faecalis*, and *Kocuria rosea*	The biomass from a bioreactor treating styrene	12 months	Commercial LDPE films	22.5% (under H_2_O_2_-biostimulation)	SEM, FTIR, AFM, TGA/DTG, GPC, GC/MS	Mohammadi et al., 2022
*Meyerozyma guilliermondii* ZJC1 and *Serratia marcescens* ZJC2	*Plodia interpunctella* larvae gut	60 days	PE films	15.87%	SEM, GC-MS	Lou et al., 2022
*Enterobacter* sp. IS2, *Enterobacter* sp. IS3, and *Pantoea* sp. IS5	Soil samples from waste disposal areas	120 days	LDPE stripes and LDPE pellets	81 ± 4 and 38 ± 3% degradation for LDPE strips and LDPE pellets	SEM, FTIR. GC-FID, tensile strength analysis	Skariyachan et al., 2016
*Aspergillus niger*, *Aspergillus flavus*, and *Aspergillus oryzae*	Purchased	55 days	LDPE bags	26.15%	SEM, FTIR	Dsouza et al., 2021
*Rhodanobacter* sp. and *Bacillus aryabhattai* 5–3	Agricultural soil	60 days	Polyethylene mulching films	–	SEM, AFM, FTIR, WCA	Wang et al., 2023

HT-GPC, high temperature gel permeation chromatography; GC-FID, gas chromatography flame ionization detector.

The current studies on the construction of functional PE degradation consortia have focused on “bottom-up” construction seeking to incorporate previously isolated strains with significant efficiency of PE degradation aiming to improve the efficiency of biodegradation significantly. Unfortunately, the lack of a clear understanding of the key functional genes, naturally occurring microbial symbioses, and metabolic mechanisms of isolated PE -degrading strains currently restrict the construction of microbial consortia through a variety of limitations, including the existence of antagonistic effects between different strains, the heavy workload associated with arbitrarily attempting different microbial combinations, and the unknown metabolic mechanisms within microbial communities. Significantly, the vast majority of microorganisms in nature cannot be directly isolated in the laboratory using traditional microbial culture technology (Kaeberlein et al., 2002). Consequently, the combination of top-down and bottom-up approaches may be able to compensate for the deficiencies of each. The top-down selective enrichment of natural microbial communities could provide a framework for reconstructing PE -degrading consortia (Gilmore et al., 2019). The application of next-generation sequencing technologies, such as (meta)genomic and (meta)transcriptomic sequencing, could enhance our understanding of microbial diversity, composition, structures, functional characteristics, and metabolic activities of natural microbial communities, thereby enabling the targeted manipulation of synthetic functional consortia structures.

### 3.3. The integration of omics analysis

In recent years, genomics, metagenomics, transcriptomics, and proteomics as well as multi omics have been increasingly applied to the study of the microbial ecology of the plastisphere and its plastic-biodegradation mechanisms due to the rapid development of high-throughput sequencing (Gravouil et al., 2017; Zhang Z. et al., 2022; Rüthi et al., 2023). Through metagenomic analysis, the genomic information of microorganisms could be retrieved directly from the landfill plastisphere and used to construct genomic libraries (Culligan and Sleator, 2016). The interpretation of the microbial community structures of the plastisphere could uncover novel genes or enzymes involved in plastic-degradation pathways (Purohit et al., 2020). Pinto et al. (2022) analyzed a metagenome of seawater-derived PE biofilms after 2 years of enrichment with LDPE as a carbon source and found that functional genes gradually became more dominant over time. They also identified numerous microorganisms, metabolic pathways, and genes that could utilize LDPE and related components, including the gene *alkb*. Hou et al. (2022) discovered that biofilm formation was a distinct metabolic pathway through their genomic analysis of two *Pseudomonas* strains with potential PE -degradation capacity. Multiple genes that could encode PE -degrading enzymes were found in both strains (Hou et al., 2022). Gravouil et al. (2017) combined transcriptomics and lipidomics to analyze the expression of functional genes associated with *R. ruber* under PE exposure. Compared to the mannitol-supplemented controls, the PE supplementation resulted in the increased expression of 158 genes in *R. ruber*, with the most upregulated pathways associated with alkane degradation and fatty-acid β-oxidation. In the recent study, Gao and Sun (2021) reported that they obtained a marine bacterial community that effectively degraded PE and PET by screening a large number of samples collected from plastic waste. They then combined next-generation sequencing techniques to gain insights into the community composition and abundance, as well as three purified bacterial strains that were reconstituted into a synthetic functional bacterial community that was similar to the native microbial community. Transcriptomics was then used to elucidate the degradation mechanisms and the expression of the upregulated genes involved in the associated enzymes.

The integration of multi-omics analysis could not only contribute to the elucidation of PE -degradation pathways, microbial abundance, and community distributions, but also effectively reveal metabolic capacities at community and individual levels (Meyer-Cifuentes et al., 2020). In addition, the real-time multi-omics analysis of synthetic functional consortia could be used to monitor the dynamics and metabolic relationships between the functional consortia and could act as a guide for the later manipulation of population ratios and environmental parameters.

## 4. The exploration and development of plastisphere

Natural microbial communities that form in ecological environments through long-term diversification, dispersion, selection, and drift have higher levels of complexity and diversity than synthetic synthetic consortia (Nemergut Diana et al., 2013). The symbiotic relationships that are established through interactions, such as reciprocity (commensalism and mutualism) and resource-based competition, help to maintain the diversity and stability of natural microbial communities (Blanchard and Lu, 2015; Liang et al., 2022; Qin et al., 2022). Correspondingly, the plastisphere is formed of natural microbial communities that are tightly attached to the surfaces of plastic debris that have been present in the natural environment for a long time. The structures of the microbial communities in the plastisphere vary depending on environmental factors, polymer type, and exposure time (Kirstein et al., 2019; Zhang S. J. et al., 2022).

The concept of the plastisphere, first proposed by Zettler et al. (2013), refers to diverse microbial communities consisting of heterotrophs, autotrophs, predators, and symbionts, which are attached to the surfaces of waste plastic materials. Through the discovery of pits on the surfaces of plastic waste debris using scanning electron microscope (SEM) techniques, it was hypothesized that the plastisphere, as a naturally occurring microbial ecological phenomenon, would have the potential to metabolize plastic waste. To date, many studies have explored the plastisphere in aquatic ecosystems such as oceans (Delacuvellerie et al., 2022b), lakes (Di Pippo et al., 2022), and rivers (Delacuvellerie et al., 2022a), as well as in soil ecosystems such as farmlands (Wang et al., 2022), mountains (Rüthi et al., 2023), and landfills (MacLean et al., 2021), using techniques such as amplicon sequencing and metagenome sequencing. These studies have analyzed the microbial community structures in the plastisphere and compared them to the microbial community structures in the surrounding environment from the same environmental source (Zhang S. J. et al., 2022; Zhurina et al., 2022). Moreover, the formation of mature and stable biofilms on the surfaces of plastic debris is required for the establishment of the plastisphere and the gradual formation of biofilms reduces the hydrophobicity of plastic and alters its functional groups (Tu et al., 2020; Du et al., 2022). With the formation of biofilms, the richness and diversity of species in the plastisphere gradually declines and the microorganisms involved in biofilm development and plastic degradation eventually come to dominate the microbial communities (Nguyen et al., 2022). In this regard, studies on biofilm formation and structure in the plastisphere may provide new ideas for consortium construction. In the construction of synthetic consortia, the existence of biofilms can stimulate the colonization of functional strains, stabilize the diversity of genotypes, and protect cells from detrimental environmental disturbances (Giri et al., 2020). Furthermore, studies on terrestrial ecosystems, especially landfills, as they are the most contaminated with plastic waste, have lagged significantly behind those on aquatic ecosystems. Since landfills are perennially contaminated with various types of plastic waste, the landfill plastisphere contains a variety of microbial community structures and potentially functional microorganisms that differ from those of marine ecosystems (Kumar et al., 2021). As a result, further research on the terrestrial plastisphere (especially in landfills, petroleum-hydrocarbon-contaminated soils, etc.) may provide new opportunities for the development of as yet unexplored potential plastic-degrading microbes/genes.

**FIGURE 4:**
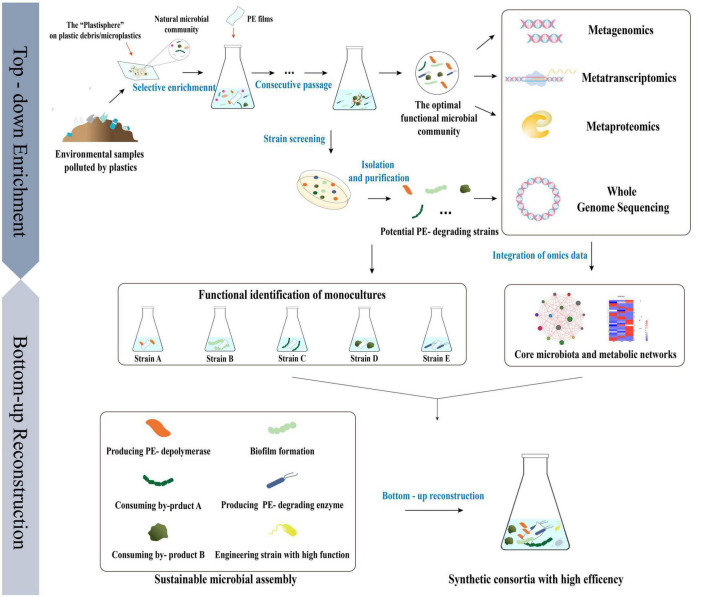
The strategy of “top-down” enrichment and “bottom-up” reconstruction combined to develop efficient PE degrading microbial consortia based on multi-omics analysis of the natural microbial community derived from the “Plastisphere” and the functional microbial community achieved after enrichment.

In general terms, top-down strategies have been used to selectively enrich the natural microbial communities of the plastisphere and optimize environmental parameters to obtain minimal and efficient plastisphere-derived PE -degrading microbial communities (Díaz-García et al., 2021). The studies to date on the metagenomics of the plastisphere and its functional microbial communities after enrichment have contributed to the identification of core and specific microbial populations and their ecological preferences, which could help to more effectively manipulate the compositions of microbial communities and comprehend the synergistic effects of strains (Zettler et al., 2013; Pinnell and Turner, 2019). Moreover, multi-omics, i.e., the combination of metagenomic, transcriptomic, and proteomic analyses, could be applied to interpret the microbial ecology of the plastisphere at the species, gene, and metabolic levels and help us to better understand the metabolic pathways that are involved in the process of PE degradation (Tiwari et al., 2022). Single strains with potential PE -degradation activities have been isolated from enriched communities and artificially reconstructed into stable and efficient synthetic functional consortia using bottom-up strategy, based on an omics analysis of the original plastisphere and its functional microbial communities, as well as a whole-genome analysis of the single strains (Figure 4; Jia et al., 2016; Liu et al., 2020).

## 5. Future perspectives toward PE biodegradation and valorization

### 5.1. Current bottlenecks and potential researches in PE biodegradation

Despite the isolation and identification of a few functional PE-degrading microbes, the biodegradation process has been relatively slow in the majority of recent studies, with microbial culturing times ranging from 30 to 120 days and only minor degradation observed using characterization techniques such as weight loss or scanning electron microscopy. The most evident limitation of present research is the lack of uniform criteria and reliable methods for precisely quantifying deterioration efficiency. Currently, the most common type of PE substrate that is utilized in investigations is commercial PE. In addition, different studies have used different PE types (powder, particles, pieces, or films), initial weights, pretreatments, measurements, and culture conditions. Therefore, it is crucial to establish standard procedures that can be used to quantify PE degradation in subsequent studies to evaluate the biodegradation ability of microorganisms (Zhang Y. et al., 2022). Commercial PE also contains additives, such as plasticizers, colorants, and antioxidants, which make PE less degradable. It is also difficult to tell whether microorganisms preferentially utilize the additives as preferred carbon sources over the plastic, resulting in false positives. In addition, it is uncertain whether the various additives have negative impacts on the process of microbial biodegradation due to their levels of toxicity. Therefore, it is recommended that further studies eliminate additives and other contaminants using pretreatment processes, such as extraction, prior to investigating biodegradation (Ferreira et al., 2022). Alternatively, the integration of strains with specific degradation additives into synthetic consortia could be effective in both degrading the various additives and resolving PE polymers in synergy with other functional bacteria in the consortia.

Moreover, the low enzyme production capacities and simple enzymatic systems of single strains, as well as the lack of discovery of efficient specific enzymes for PE degradation in studies to date, render single strains incapable of effectively tackling the complex PE -degradation process. Therefore, it is suggested that both the exploration of novel efficient PE-degrading enzymes/genes and the investigation of degradation mechanisms be strengthened in future research. Additionally, the combination of genetic engineering and enzyme engineering technologies could help with the engineering of efficient PE-degrading-enzyme-producing bacteria and the enhancement of related enzyme activities. Various abiotic (e.g., temperature, UV, dissolved oxygen, humidity, pH, etc.) and biotic (e.g., microbial diversity and abundance, microbial enzymatic activity, etc.) factors affect the process of microbial PE degradation (Maity et al., 2021). Thus, different factors, including environmental parameters and culture conditions, should be considered to establish the optimal conditions for PE biodegradation.

As the production and properties of byproducts from PE -biodegradation pathways are not currently fully understood, there is an urgent need for additional studies on the properties of these byproducts and their implications for later-stage fermentation and environmental toxicity.

Remarkably, the majority of previous studies have focused on the degradation of large pieces of PE. In contrast, there has been a lack of studies on PE micro/nanoplastics, which are relatively tiny in size and well-hidden in the environment. These micro/nanoplastics are not as easily collected and disposed of as large pieces of plastic and, as they absorb enormous quantities of dangerous compounds during their movements around the environment, they significantly enhance the difficulty of plastic degradation (Liu et al., 2022a). For this reason, there is still a need to further investigate collection methods for and the biodegradation of PE micro/nanoplastics in the environment.

The microbial community structures and associated biofilms of the plastispheres in different ecosystems polluted by plastic waste have not been fully explored, particularly those in terrestrial ecosystems; hence, comprehensive multi-omics studies should be conducted to reveal the biodiversity, biofilm-formation mechanisms, and biodegradation capacity of these plastispheres in the future. Additionally, microbial metabolic engineering based on recombinant DNA or gene-editing technology may be effective to some degree in solving the limitations of PE-degrading single strains, such as poor degradation ability and low enzyme production capacity, thereby enhancing the metabolic activities of PE -degrading strains and significantly enhancing their biological degradation efficiency (Sharma and Shukla, 2022). Engineered microorganisms with high efficiency for degrading PE that are obtained through recombinant DNA technology or gene editing may have potential for future large-scale industrial applications.

### 5.2. The potential approaches for PE-valorization

Especially remarkable is the fact that, with the severe damage to the environment caused by conventional plastic disposal methods, more economically and environmentally acceptable plastic valorization is now increasingly being proposing and developed to address the ever-increasing volume of plastic waste. The valorization of waste plastics aims to maximize the value of plastic waste by recycling, upcycling, biodegradation, or energy recovery to dispose of the plastic waste in more sustainable ways and/or to transform them into brand new and functional products (Hou et al., 2021; Zhang F. et al., 2022; Table 4). Residual bulk plastic waste can be processed by either mechanical or chemical recycling, which involves manufacturing waste plastics into homogeneous or homologous renewable plastic products or breaking them down into their constituent molecules and applying them to produce other products, such as fuels, chemicals, etc. (Ragaert et al., 2017). Nowadays, the most common and time-efficient method of recovering energy from waste plastics is incineration, whereby part of the heat generated by the incineration process is recovered and applied to heat or electricity generation (Damayanti et al., 2022). Unfortunately, conventional recycling or directly incinerating plastic waste has many shortcomings, namely the labourious processes of collection, sorting and cleaning, the finite recycling times, exorbitant costs, the emission of hazardous residues and the unsuitability for heavily weathered plastic debris or micro/nano plastics (Lomwongsopon and Varrone, 2022). Instead, upcycling of plastic waste via chemical or biological methods into smaller molecules and transforming these molecules into new and valuable products is gradually appearing to be an attractive and more eco-friendly route to valorizing plastics in order to maximize the value of post-consumer plastics while minimizing the amount of plastic waste being directly discarded into the environment (Hou et al., 2021). The current studies on the upcycling of PE and other plastic wastes mainly focuses on the production of plastic wastes into fuels, high-value chemicals and multifunctional materials by chemical reactions including thermochemical catalysis (hydrolysis, oxidation, tandem catalysis, and carbonization), electrocatalysis, and photocatalysis, which will be widely applied in various fields such as energy, chemicals, medicine, construction, and textiles (Zhou et al., 2022).

**TABLE 4:** Potential products and application area of waste plastic valorized via different strategies and technical routes.

The forms of valorization	Tethnological processes		Potential products	Application fields	References
Recycling	Primary (closed-loop) recycling		New plastic products	–	Hou et al., 2021
	Secondary (mechanical) recycling				
	Tertiary (chemical) recycling	Pyrolysis, gasification, and depolymerization, etc.	Gases, liquids (olefins, alkanes and other hydrocarbon compounds, gasoline), waxes, lubricants, monomers or oligomer	Energy resources, chemical feedstock	Lee et al., 2021
Upcycling	Chemical upcycling	Thermochemical catalysis	Lightweight tiles, Plastic lumber, Bricks and blocks	Construction materials	Dhawan et al., 2019; Awoyera and Adesina, 2020
			Liquids (naphtha, lubricants), chemicals (long-chain alkyl aromatics) and solid waxes	Chemical feedstock, fuels	Zhang F. et al., 2020; Dai et al., 2021; Jia et al., 2021
			Pollutants adsorbent	Wastewater Treatment/Environmental remediation	Zhang H. et al., 2020
			Surface coatings for commercial fabrics	Textile	Wu et al., 2022
			Combined materials (porous carbon nanosheets, graphene foil, zwitterionic hydrogel), carbon materials, new polymers, composite materials	Functional materials	Gong et al., 2014; Zhuo and Levendis, 2014; Cui et al., 2017; Ayana et al., 2022; Yue et al., 2022a,b
			Gas or liquid fuels (H_2_, bio-oil, bio-crude oil, synthesis gas)	Energy resources	Nanda and Berruti, 2021
		Photocatalysis	Fuels (H_2_), chemicals (HCOOH, C_2_H_4_, C_2_H_6_, CH_3_COOH), and new polymers	Energy resources, chemical feedstock, functional materials	Zhou et al., 2022
		Electrocatalysis	Modified polymers, H_2_, organic acids, hydrocarbons	Energy resources, chemical feedstock, functional materials	Zhou et al., 2022
	Biological upcycling	living organisms/enzymes	Biofuels (ethanol, hexadecanoate, methane)	Energy resources	Gluth et al., 2022
			Chemicals (succinic acid, wax esters, fatty acids, biosurfactants)	Chemical feedstock, Food industry, pharmaceuticals, cosmetics	Ru et al., 2020; Gregory et al., 2023
			Bio-based materials polyhydroxyalkanoate	Packaging, agriculture, and medical materials	Guzik et al., 2014
			Proteins, peptides, and amino acids	Animal feed	Sangiorgio et al., 2021; Schaerer et al., 2023
Biodegradation	Biodepolymerization	Invertebrates, microorganisms, and enzymes	H_2_, CH_4_, alkanes, fatty acids	Fuels, platform chemicals	Lomwongsopon and Varrone, 2022
	Compost		Biomass, organic matter	Energy resources, fertilizer	Millican and Agarwal, 2021
Energy recovery	Incineration		Thermal energy	Power supply for domestic or business use	Damayanti et al., 2022

**FIGURE 5:**
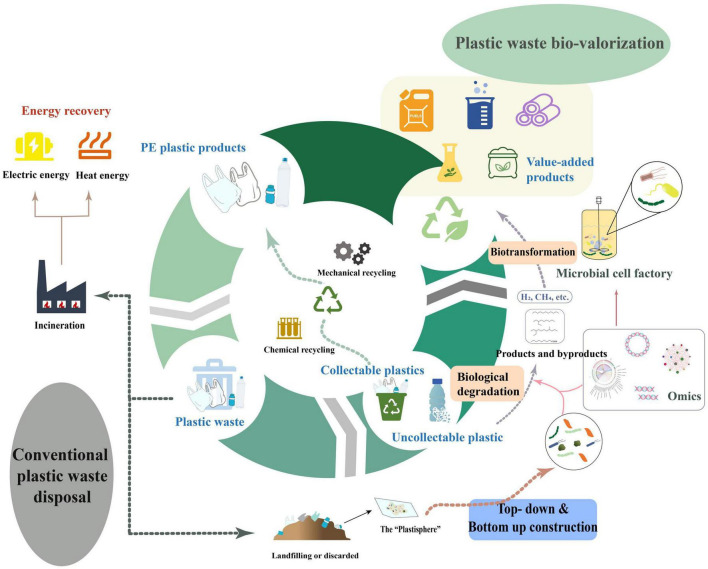
A general overview of plastic waste bio-upcycling.

The bio-upcycling of plastic waste is more sustainable and reaction conditions are more moderate than chemical processes. Combining biodegradation and upcycling of plastic waste enables to treat the non-recyclable plastic waste in the environment as well as to overcome the issue of product and by-product treatment during degradation of plastics. For one thing, the by-products and products derived from the biodegradation of PE could be taken directly into fuels or platform chemicals such as hydrogen, methane and various alkanes, fatty acids, etc. For the other hand, the oligomers or monomers obtained from the biodepolymerization of plastic can be biotransformed by engineered microbial cell factories to produce various value-added products (e.g., biofuels, fine chemicals, bio-based materials, biosurfactants, etc.) by upcycling (Guzik et al., 2014; Ru et al., 2020; Hou et al., 2021; Jehanno et al., 2022; Figure 5). Significantly, there is still relatively few studies on the upcycling of plastic waste, especially polyolefins such as PE, by microorganisms or enzymes given that there is limited understanding around the mechanisms of plastic biodegradation. The strategy of integrating microbial consortiums with multi-omics can further suggest a new vision on the bio-upcycling of plastic waste. The application of multi-omics will contribute to the deeper insights on the metabolic mechanisms of plastic biodegradation and the understanding of microbial interactions between microbial communities. The rational design and synthesis of the microbial consortia’s composition and the pathways of microbial metabolism would be more favorable to the promising biotransformation into a larger variety of potential products of plastic biodegradation, thereby extending the range of products that can be produced through upcycling of plastic waste.

Furthermore, the adoption of gene engineering, metabolic engineering and other technologies allow further enhancement of functional strains and optimization of metabolic pathways for more efficient and targeted biotransformation into different products or specific chemicals derived from waste plastics (Sullivan et al., 2022).

## 6. Conclusion

The prevalence of plastic waste and micro/nanoplastic pollution has always been a great challenge facing environmental remediation. Traditional plastic disposal methods have not been effective in solving the global plastic waste pollution problem, which is becoming increasingly serious. However, recent studies on plastic biodegradation have led to new perspectives on the sustainable disposal of plastic waste and the discovery of an increasing number of microbes and other invertebrates that are involved in plastic degradation. PE is the most widespread and recalcitrant petroleum-based plastic and the study of its biodegradation has received considerable interest over the years. The microorganisms and other organisms involved in the degradation of PE have been extensively explored in the literature; however, there have been no breakthroughs in the study of PE biodegradation, particularly regarding the mechanisms of PE degradation and the identification of specific PE depolymerases. Recent studies have examined the constraints that single strains encounter when trying to degrade PE, and progressive efforts have been made to combine several strains to develop more effective consortia or co-cultures. Compared to single strains, functional microbial consortia offer many advantages in degrading plastic waste; however, the lack of an understanding of the byproducts and mechanisms of PE degradation has limited the development of artificial functional consortia for PE degradation, which was the focus of this review.

Considering the existing bottlenecks in the construction of PE-degrading consortia and the pros and cons of each strategy, we propose combining top-down and bottom-up approaches for the synthesis of efficient PE-degrading consortia. In this way, the selective, top-down enrichment of natural microbial communities can be applied to optimize community functions, and the enriched communities can undergo omics analyses of their functions and community structures to help us better understand the microbial PE -degradation mechanisms and microbial interactions at play within the communities. The insights into microbial communities achieved using top-down strategies could contribute to a more reasonable and appropriate bottom-up synthesis of functional PE -degrading consortia. The plastisphere is a new ecological niche that spontaneously forms on plastic debris and is closely associated with plastic polymers. Investigating the microorganisms or microbial communities that grow in the plastisphere could uncover principal sources of novel PE-degrading microorganisms/microbiota. The combination of genomic, transcriptomic, metagenomic, and other omics/meta-omics analyses could help to unravel the microbial diversity and community composition of the plastispheres in different ecosystems and the metabolic relationships between microbes, which could also enable the discovery of core microbiota and key pathways/genes for PE degradation and provide a foundation for the subsequent construction of functional consortia.

We close with a discussion of concerns and bottlenecks in the existing studies on PE biodegradation as well as some insights into the topics that should be considered in future research. Due to insufficient study of the mechanisms of PE biodegradation, the construction of effective microbial consortia currently faces numerous obstacles. In addition, the safety and stability of synthetic microbial consortia and the regulation of coexistence between microorganisms could represent challenges for future research due to the dynamic nature of microbial communities, biological variability, and various other uncertainties.

## Author contributions

CZ: conceptualization, data curation, and writing-original draft preparation. YM, TL, and F-JJ: data curation and visualization; C-ZJ and H-MO: resources and visualization; H-GL. and LJ: supervision and writing-reviewing and editing. All authors have read and agreed to the published version of the manuscript.
